# The HLA class I immunopeptidomes of AAV capsid proteins

**DOI:** 10.3389/fimmu.2023.1212136

**Published:** 2023-08-16

**Authors:** Carlos A. Brito-Sierra, Megan B. Lannan, Laurent P. Malherbe, Robert W. Siegel

**Affiliations:** Lilly Research Laboratories, Eli Lilly and Company, Indianapolis, IN, United States

**Keywords:** aav, HLA class I, gene therapy, CD8+, proteomics

## Abstract

**Introduction:**

Cellular immune responses against AAV vector capsid represent an obstacle for successful gene therapy. Previous studies have used overlapping peptides spanning the entire capsid sequence to identify T cell epitopes recognized by AAV-specific CD8+ T cells. However, the repertoire of peptides naturally displayed by HLA class I molecules for CD8 T cell recognition is unknown.

**Methods:**

Using mRNA transfected monocyte-derived dendritic cells (MDDCs) and MHC-associated peptide proteomics (MAPPs), we identified the HLA class I immunopeptidomes of AAV2, AAV6 and AAV9 capsids. MDDCs were isolated from a panel of healthy donors that have diverse alleles across the US population. mRNA-transfected MDDCs were lysed, the peptide:HLA complexes immunoprecipitated, and peptides eluted and analyzed by mass spectrometry.

**Results:**

We identified 65 AAV capsid-derived peptides loaded on HLA class I molecules of mRNA transfected monocyte derived dendritic cells. The HLA class I peptides are distributed along the entire capsid and more than 60% are contained within HLA class II clusters. Most of the peptides are organized as single species, however we identified twelve clusters containing at least 2 peptides of different lengths. Only 9% of the identified peptides have been previously identified as T cell epitopes, demonstrating that the immunogenicity potential for the vast majority of the AAV HLA class I immunopeptidome remains uncharacterized. In contrast, 12 immunogenic epitopes identified before were not found to be naturally processed in our study. Remarkably, 11 naturally presented AAV peptides were highly conserved among the three serotypes analyzed suggesting the possibility of cross-reactive AAV-specific CD8 T cells.

**Discussion:**

This work is the first comprehensive study identifying the naturally displayed HLA class I peptides derived from the capsid of AAVs. The results from this study can be used to generate strategies to assess immunogenicity risk and cross-reactivity among serotypes during gene therapies.

## 1 Introduction

Gene therapies are being widely used to treat diseases that would be uncurable with traditional medicine. One of the main strategies to deliver gene therapies are the adeno-associated viruses (AAVs). These viruses are attractive vectors because they can be engineered to deliver a nucleic acid of interest with low risk of genome insertion and no replication in the patient (1–3). There are currently three FDA approved therapies that utilize AAVs as the delivery platform: Luxturna, Zolgensma and Hemgenix (4, 5). One drawback to the use of AAV-mediated therapy stems from immunological response.

The risk that immunogenicity poses on AAV-based gene therapies has been demonstrated in hemophilia clinical trials: participants were shown to have anti-drug immunity and resulted in reduced expression of factor IX (6, 7). Immunogenicity can be the result of a combination of innate, humoral and cellular immunity. For gene therapies, cellular immunity is particularly concerning because activated CD8+ T cells can recognize the cells that have been transduced with AAV and lyse them. This can lead to elimination of the transduced gene and in some cases lead to tissue damage and organ failure (8–12).

CD8+ T cells recognize peptides presented in the context of HLA class I molecules. Previous studies have mapped CD8+ T cell epitopes using overlapping peptide pools spanning the entire capsid of AAVs and bioinformatic algorithms (13–16). Some peptides have been validated with MHC multimer staining (6, 17), including one of the most recognized immunogenic epitopes of AAV2, VPQYGYLTL (6, 18) and its cross-reactive homolog peptide in other serotypes, IPQYGYLTL (19). However, the repertoire of naturally displayed HLA class I peptides remain unknown. MHC-associated peptide proteomics (MAPPs) is a tool that enables the identification of naturally presented peptides in both HLA class I and class II molecules through a multi-step process involving the cultivation of human primary antigen-presenting cells, peptide isolation, and subsequent identification using liquid chromatography-mass spectrometry (LC-MS) (20–22). We previously used this method to elucidate the HLA class II immunopeptidome from different AAV serotypes (22). However, MAPPs has not been previously implemented to identify the AAV capsid derived peptides naturally presented on HLA class I molecules.

In our current study, we identified the naturally processed and presented HLA class I immunopeptidomes of AAV2, AAV6 and AAV9 capsids using mRNA-transfected monocyte-derived dendritic cells (MDDCs) from a panel of donors representing a broad range of alleles. We identified peptides distributed along the entire capsid sequences and examined how they compare with previous studies. Our study also highlights sequence conservation of several HLA class I peptides among the three serotypes and describes overlap with the previously identified HLA class II immunopeptidomes. Combined, these results represent the first comprehensive study of the HLA class I immunopeptidomes of AAV capsids and provides peptides that should be considered for pre-existing immunogenicity risk assessment to AAV-based gene therapies.

## 2 Materials and methods

### 2.1 Isolation and differentiation of monocyte derived dendritic cells

Buffy coats were obtained from American Red Cross with written consent from healthy donors. For isolation of monocytes, the protocol from Karle 2020 (20) was adapted as follows: buffy coats were diluted 1:1 with PBS and transferred to a Ficoll-Pacque density gradient medium (GE HealthCare #17544203) in SepMate tubes (Stem Cell Technologies #86450). Tubes were centrifuged for 10 minutes at 700 x g at 20°C. Three layers were formed: a plasma layer, a PBMC interphase and the layer of Ficoll-pacque. The PBMC layer was transferred to another tube and centrifuged for 10 minutes at 700 x g at 20°C. The pellet was resuspended in 8 mL of chilled autoMACS^®^ Running Buffer (Miltenyi Biotec #130-091-221). To isolate CD14+ cells, 1.5 mL of CD14 microbeads (Miltenyi Biotec # 130-050-201) was added to each cell suspension and incubated for 20 minutes at 4°C. Cell suspension was washed in 40 mL of autoMACS^®^ Running Buffer and centrifuged at 300 x g for 10 minutes at 4°C. Cell pellets were resuspended in 6 mL of autoMACS^®^ Running Buffer and loaded on the AutoMACS instrument for positive selection. 300 µL of the CD14 depleted cells were sent for HLA typing in at CD genomics (https://www.cd-genomics.com/). The CD14+ cells were counted and measured for viability on the Countess II instrument (Invitrogen AMQAX1000) using trypan blue (Invitrogen #T10282). Cells were pelleted down and dissolved in growth media at a density of 1 x 10^6^ cells/mL. Growth media consisted of RPMI 1640 + Glutamine medium (Gibco #11875-093) supplemented with 5% serum replacement (Thermo Fisher Scientific #A2596101), 5 mM HEPES (Gibco #15630-080), 1% of MEM nonessential amino acids (GIBCO #11140-050), 100 U/mL Penicillin/Streptomycin (Hyclone #SV30010), 1 mM Sodium Pyruvate (Gibco # 11360070), 50 µM β-mercaptoethanol (Fisher chemical #O3446I-100), and 3.5% of DMEM high glucose (Gibco #31053-028). To induce differentiation into immature dendritic cells, growth media was also supplemented with 40 ng/mL of granulocyte-colony stimulating factor (Human GM-CSF; Sargramostim, Sanofi-Aventis, NDC #0024-5843-05) and 20 ng/mL of IL-4 (R&D Systems, #204-IL). 5 mL of cell suspension were seeded per well in 6-well plates and incubated at 37°C in an atmosphere of 5% CO_2._

### 2.2 mRNA, transfection and harvesting of MDDCs

mRNA was synthesized by TriLink Biotechnologies (https://www.trilinkbiotech.com/). The mRNA consisted of a codon optimized sequence coding for the full length VP1 capsid protein of AAV2, AAV6 and AAV9. The VP1 protein was chosen because it contains all the capsid sequences in the viral capsids. The mRNA construct also encoded for a C-terminal HiBiT tag (VSGWRLFKKI) used to determine protein expression. The modifications of the mRNA included a capped (cap1) using Cleancap^®^ AG and fully substitution with N1-Methyl-Pseudo-U.

Four days after isolation, immature monocyte-derived dendritic cells (iMDDCs) were transfected with the corresponding mRNA using Lipofectamine MessengerMAX (Invitrogen # LMRNA015) following manufacturer instructions. For the kinetic expression analyses, iMDDCs were transfected in a 96-well plate format and bioluminescence was measured with the Nano-Glo^®^ HiBiT Lytic Detection System (Promega # N3030) using the BioTek Cytation 5 instrument. Protein quantification was determined by a standard curve generated using the HiBiT control protein (Promega # N3010). Protein expression was measured at 0, 2, 4, 6 8, 24, 48, 72 and 96 hours post transfection.

For the MHC-associated peptide proteomics assays, the mRNA transfections were performed in 6-well plate format. Cells were lysed at six hours post transfection (or at the specified time points for the kinetic assay) with 0.5 mL of RIPA buffer (Thermo Fisher #89900) containing 10 units/µL DNase (Roche # 04716728001) and 1 tablet of EDTA free protease inhibitor cocktail (Roche #11836170001) per every 10 mL of lysis buffer. Two wells transfected with the same mRNA were pooled together for a final lysis volume of 1 mL. Harvested samples were stored at -80°C.

### 2.3 HLA class I peptides isolation

Immunoprecipitation of HLA molecules was performed using the Agilent AssayMap instrument as described before. Briefly, 100 µg of biotinylated anti-pan HLA class I (W6-32, produced in house) were immobilized on streptavidin cartridges (Agilent, G5496-60010) by passing over the cartridge at 5 µL/minute and washing three times with PBS. Cell lysates were thawed and filtered using a 0.2 µm hydrophilic filter plate (Analytical Sales & Services #96432-10) and loaded in a 96 well polypropylene plate (Thermo Scientific #AB1127). Filtered lysate was passed through the antibody loaded cartridges at 5 µL/minute at room temperature. Following that, the cartridges were washed twice with 50 mL of 100 mM ammonium acetate and once with 50 µL of water at 25 µL/minute. The HLA:peptide complexes were eluted with 50 µL of 5% acetic acid in 0.1% TFA at 2 µL/minute. Eluted samples were centrifuged in 10K MWCO spin filters (MilliporeSigma #MRCPRT010) equilibrated with BSA, angiotensin-I and acetic acid. 20 µL of the filtered samples were loaded in a 96-well polypropylene PCR plate. From this volume, 18 µL of isolated peptides were injected into the LC-MS system. Notably, the injection volume corresponds to the peptides originating from an estimated 3.6 million cells.

### 2.4 Liquid chromatography – mass spectrometry analysis of HLA class I derived peptides

A Thermo easy-nLC 1200 system coupled to a Q-Exactive (HFX) orbitrap mass spectrometer (Thermo Scientific) was used to analyze the eluted peptides. Separation was performed with a 75 µm x 150 mm EASY-Spray HPLC column (Thermo Scientific #ES900) coupled to a standard EASY-Spray source with an electrospray potential of 1.9 kV. The solvents used were 0.1% formic acid in water (buffer A) and 0.1% formic acid in 80% acetonitrile (buffer B). A 65 minute gradient was performed using a flow rate of 250 nL/minute as follows: 60 minutes at 2-45% of B, followed by 1 minute of 45-95% of B and finally holding at 95% of B for 4.5 minutes. The Q-Exactive was run with a full scan of 120,000 resolution in the orbitrap followed by a top 20 data dependent MS/MS cycle comprised of orbitrap scans where +2, +3 and +4 ions were fragmented with HCD (CE of 25 and 30).

### 2.5 LC-MS data analysis

RAW data files were analyzed in PEAKS online (Bioinformatic solutions) using a protein sequence fasta file containing 20606 human Uniprot entries downloaded in August 2022, plus the capsid sequence of AAV2, AAV6 and AAV9. No enzyme specificity was set, peptide mass error tolerance was set at 10 ppm for precursors and 0.03 Da for MS2 fragments. Additionally, post translational modifications were identified using the PEAKS PTM built *de novo-*led for 925 modifications. A 1% false discovery rate (FDR) was calculated using PEAKS decoy search.

### 2.6 Quantification and statistical analysis

Data manipulation was performed in KNIME 4.7.0. Analysis in KNIME included filtering for peptides specific to AAV, mapping the peptides to the corresponding AAV serotype, and summarizing number of donors per peptide, counts per peptide length, number of peptides per donor and counts of non-AAV peptides. Data representation in bar graphs and heatmaps was performed in GraphPad Prism 9, as well as t-test statistical analysis. HLA allele predictions were performed using MHCMotifDecon – 1.0 (23). The US frequency value for individual peptides was calculated using the individual frequency of the predicted HLA alleles. Tables and data curation were performed on Excel and venn diagrams were manually drawn in Power Point.

## 3 Results

### 3.1 Method development for identifying AAV capsid-derived HLA class I peptides in monocyte derived dendritic cells

**Figure 1:**
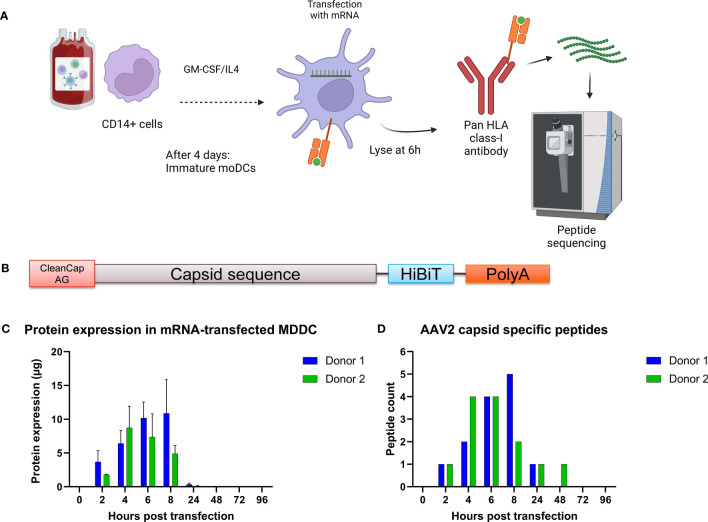
Strategy to identify AAV capsid HLA class I peptides in immature monocyte derived dendritic cells. **(A)** Schematic representing the PBMC isolation, cell differentiation into dendritic cells, mRNA transfection, HLA:peptide complex immunoprecipitation and mass spectrometry peptide sequencing. Created with BioRender.com. **(B)** Schematic of the mRNA construct. **(C)** Time course expression after transfection of monocyte derived dendritic cells with mRNA coding for the capsid of AAV2. Protein expression was determined by the HiBiT lytic detection system. **(D)** Time course identification of AAV capsid peptide on HLA class I molecules after transfection with mRNA.

To identify the peptides loaded on HLA class I molecules, we transfected mRNA coding for the VP1 capsid proteins of AAV2, AAV6 and AAV9 into monocyte-derived dendritic cells (MDDCs) as they provide access to HLA allele diversity while maintaining a similar mRNA transfection protocol across different samples. VP1 was chosen as it contains all the sequences of the AAV capsid. After mRNA transfection of MDDCs, HLA:peptide complexes were immunoprecipitated and the peptides were eluted and analyzed by liquid chromatography-mass spectrometry and identified using PEAKS (**Figure 1A**). To determine the optimal time point after mRNA transfection to detect AAV capsid peptides, we performed a time course expression study using MDDCs from two donors. The mRNA construct encodes for a AAV2-VP1 capsid labeled at the C-terminal with the HiBiT sequence (**Figure 1B**), used for a luciferase-based assay to detect protein expression. We observed that the highest protein expression (**Figure 1C**) and the highest number of unique capsid specific peptides was detected at 6 hours post transfection (**Figure 1D**). The results for this section set the basis for the rest of the study, where the identification HLA class I immunopeptidomes of AAV capsids was performed at 6 hours post mRNA-transfection of MDDCs.

### 3.2 Profiling HLA class I peptides in monocyte derived dendritic cells transfected with mRNA coding for the capsids of AAV2, AAV6 and AAV9

**Figure 2:**
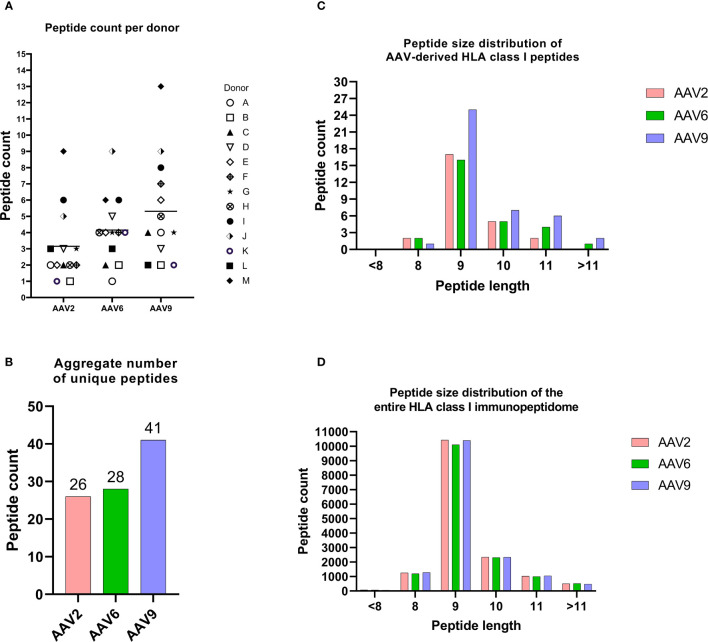
The HLA class I immunopeptidomes of AAV serotypes. **(A)** Peptide counts per donor and serotype. **(B)** Aggregate number of unique peptides for each AAV serotype. **(C)** Peptide size distribution of AAV capsid-derived HLA class I peptides. **(D)** This figure illustrates the distribution of aggregate peptide sizes across the entire immunopeptidome of cells transfected with various mRNAs. The peptide counts depicted in this figure represent unique and non-repetitive peptides.

To characterize the HLA class I immunopeptidome of AAV capsid proteins, we transfected MDDCs from 13 healthy donors with mRNA coding for the capsid protein of AAV2, AAV6 and AAV9. These serotypes were chosen because they are being widely used in clinical trials. The 13 donors had 36 HLA alleles that cover 71.7%, 55.4% and 72.5% frequencies in the US population for the HLA-A, HLA-B and HLA-C locus, respectively (**Table 1**). Across the 13 donors and 3 serotypes, we detected 65 different AAV capsid peptides presented by transfected MDDCs. The total AAV peptide numbers varied among donors and serotype, with AAV9-transfected MDDCs displaying more peptides (41 peptides) than AAV2 (26 peptides) and AAV6 (28 peptides) (**Figures 2A, B**, **Supplemental Table 1**). The peptides were assigned to an HLA allele based on a peptide affinity prediction. Most peptides were predicted to bind HLA-B (43 peptides), whereas fewer peptides were predicted to bind HLA-A (15 peptides) and HLA-C (7 peptides) (**Supplemental Figure 1**). The majority of the peptides displayed (59 out of 65) have not been described before. The AAV specific peptides had a classical peptide length distribution of HLA class I, where most peptides are 9 amino acid residues long (**Figure 2C**). Although HLA class I molecules typically bind peptides 8-10 aa long, 23% of the peptides identified by MAPPS (15 peptides) ranged in size from 11aa (12 peptides) to 12-13 aa (3 peptides). The total number of non-AAV HLA class I peptides in mRNA transfected cells was similar among the three treatments, suggesting that the differences in AAV specific peptides is not the result of differential cellular stress conditions (**Figure 2D**).

**Table 1:** Donor HLA alleles and frequencies in the US population.

	HLA-A		HLA-B		HLA-C	
Donor	Alleles	US Frequency	Alleles	US Frequency	Alleles	US Frequency
A	02:01, 23:01	26.70%	07:02, 49:01	12.20%	07:01, 07:02	26.70%
B	01:01, 03:01	23.80%	07:02, 08:01	19.50%	07:01, 07:02	26.70%
C	02:05, 11:01	6.60%	15:01, 50:01	5.80%	03:04, 06:02	15.70%
D	01:01, 11:01	18.00%	35:01, 51:01	10.20%	04:01, 15:02	14.40%
E	02:01, 02:01	23.60%	15:01, 44:02	11.20%	03:04, 05:01	14.30%
F	02:01, 24:02	32.50%	08:01, 27:05	11.40%	02:02, 07:01	18.00%
G	11:01, 11:01	5.50%	07:02, 35:57	10.50%	04:01, 07:02	25.30%
H	01:01, 02:01	36.10%	08:01, 40:01	13.30%	03:04, 07:01	21.30%
I	32:01, 68:01	5.60%	40:02, 44:02	8.10%	02:02, 07:04	5.20%
J	01:01, 02:01	36.10%	40:01, 57:01	7.00%	03:04, 06:02	15.70%
K	02:01, 11:01	29.10%	40:01, 56:01	4.70%	01:02, 03:04	11.00%
L	01:01, 01:01	12.50%	15:17, 50:01	1.30%	06:02, 07:01	22.10%
M	02:01, 03:01	34.90%	27:05, 35:01	8.10%	02:02, 04:01	16.50%
Total		71.70%		55.40%	Total	72.50%

HLA class I haplotype frequency data was retrieved from the National Marrow Donor Program for HLA-A (https://bioinformatics.bethematchclinical.org/workarea/downloadasset.aspx?id=6372), HLA-B (https://bioinformatics.bethematchclinical.org/workarea/downloadasset.aspx?id=6386) and HLA-C (https://bioinformatics.bethematchclinical.org/workarea/downloadasset.aspx?id=6390). These frequencies were then multiplied by the relative percentage of ethnic groups in the U.S. population based on 2020 census values representing overall frequencies (https://www.census.gov/quickfacts/fact/table/US/PST045222). Unique allele frequencies were added up to calculate the total frequency of the alleles in our study.

**Figure 3:**
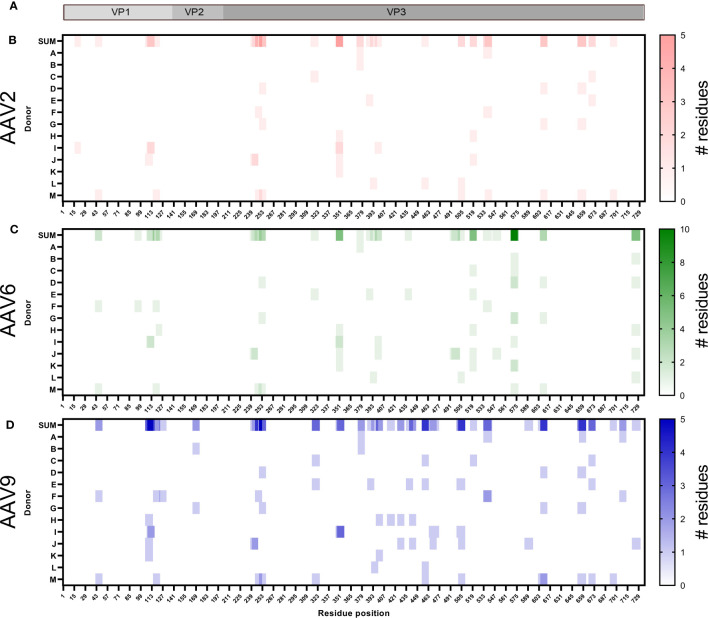
The HLA class I peptides of AAV are distributed along the entire capsid protein. **(A)** Diagram representation of the full-length sequence of VP1, highlighting the regions that correspond to VP1 unique, VP1/VP2 unique and VP3. **(B–D)** Heatmap representation of the AAV2 **(B)**, AAV6 **(C)** and AAV9 **(D)** HLA class I peptides identified. The first row, shown as SUM, represents the sum of the times a residue was displayed among multiple donors in different or identical peptides. Rows A to M represent the donor IDs as shown in **Table 1**. White background represents no peptide identified whereas the intensity of the colors, red (AAV2), green (AAV6) or blue (AAV9), provides information about the number of instances in which a particular residue is contained in an identified peptide. For instance, a single peptide composed of residues 42-50 would be designated with the lightest scale from each donor in which it was identified. If overlapping peptides were identified from the same donor (e.g. residues 107-116 and 108-116), then the area of overlap would be a darker shade of color. The X-axis represents the residue position in the capsid of the corresponding AAV.

The identified peptides were aligned to the corresponding sequence of the AAV serotype and represented as heatmaps (**Figure 3**). We found HLA class I peptides covering 25%, 26% and 37% of the capsid protein of AAV2, AAV6 and AAV9, respectively (**Figures 3B-D**). The individual peptides are dispersed along the capsid without displaying any noticeable aggregations in particular regions. The peptide sequences are shown in **Supplemental Tables 2–4** along with other analyses including predicted MHC allele and number of donors with a particular allele presenting the peptide. As expected, we observed that the majority of the immunopeptidomes were organized as single peptide species. However, we identified 12 clusters containing at least 2 peptides of different lengths (**Table 2**). Clusters 1 and 2 are identical between the three AAV serotypes and cluster 3 is identical between AAV2 and AAV6.

**Table 2:** HLA class I clusters of AAV capsids.

Cluster	Cluster peptides													Length	Serotype	Donors	HLA alleles
1	D	T	S	F	G	G	N	L	G	R				10	AAV2 AAV6 AAV9	I	HLA-A68:01
		T	S	F	G	G	N	L	G	R				9		I	HLA-A68:01
2	R	V	I	T	T	S	T	R	T	W				10	AAV2 AAV6 AAV9	J	HLA-B57:01
			I	T	T	S	T	R	T	W				8		J	HLA-B57:01
3	D	S	E	Y	Q	L	P	Y	V	L				10	AAV2 AAV6	I	HLA-B40:02
		S	E	Y	Q	L	P	Y	V	L				9		H, I, J, K	HLA-B40:01, HLA-B40:02
4	T	D	S	D	Y	Q	L	P	Y	V	L			11	AAV9	I	Undefined
		D	S	D	Y	Q	L	P	Y	V	L			10		I	Undefined
			S	D	Y	Q	L	P	Y	V	L			9		I	HLA-B40:02
5	S	V	A	G	P	S	N	M	A	V	Q	G	R	13	AAV9	I	HLA-A68:01
		V	A	G	P	S	N	M	A	V				9		J	HLA-C03:04
6	K	T	K	T	D	N	N	N	S	N	F	T	W	13	AAV6	J	HLA-B57:01
			K	T	D	N	N	N	S	N	F	T	W	11		J	HLA-B57:01
7	S	E	F	A	W	P	G	A	S	S	W			11	AAV9	E, I	HLA-B44:02
			F	A	W	P	G	A	S	S	W			9		J, M	HLA-B57:01, HLA-B35:01
8	K	F	F	P	Q	S	G	V	L	I	F			11	AAV2	A, F	HLA-A23:01, HLA-A24:02
			F	P	Q	S	G	V	L	I	F			9		M	HLA-B35:01
9	R	F	F	P	L	S	G	S	L	I	F			11	AAV9	A, F	HLA-A23:01, HLA-A24:02
	R	F	F	P	L	S	G	S	L	I				10		F	HLA-A24:02
10	A	T	N	P	V	A	T	E	R	F				10	AAV6	B, C, D, G, K, M	HLA-A01:01, HLA-A11:01, HLA-B15:01, HLA-C02:02
	A	T	N	P	V	A	T	E	R					9		D, G, I, K	HLA-A11:01, HLA-A68:01
11	L	P	G	M	V	W	Q	D	R	D	V	Y		12	AAV9	M	HLA-B35:01
				M	V	W	Q	D	R	D	V	Y		9		D, M, G	HLA-B35:01, HLA-B35:57
12	T	P	V	P	A	D	P	P	T	A	F			11	AAV9	D, M, G	HLA-B35:01, HLA-B35:57
			V	P	A	D	P	P	T	A	F			9		A	HLA-B07:02

Our analyses identified three different types of clusters based on the presentation of peptides by donors and their predicted binding to different alleles. The first type of cluster matches the traditional definition where multiple peptides are presented by the same donor, and Cluster 1 is an example of this type. The second type of cluster contains singletons, which are peptides of varying lengths found in different donors that are predicted to bind to different alleles but overlap when considered in a group. An example of this type is Cluster 5, where a 13-mer peptide is predicted to bind to HLA-A68:01 and a 9-mer peptide to HLA-C03:04. The third type of cluster is a mixture of the first two types and contains peptides of varying lengths from the same donor, with one or more of those peptides being presented as singletons in other donors. Cluster 3 is an example of this type, where a 10-mer peptide was found in one donor and predicted to bind to HLA-B40:02, while a 9-mer peptide was found in several donors and predicted to bind to both HLA-B40:01 and HLA-B40:02. Additional details on these clusters can be found in **Table 3**.

**Table 3:** Naturally processed HLA class I peptides and their match with previous identified epitopes.

MAPPs peptide	Serotype	HLA found in this study	HLA described in previous studies	References
VPQYGYLTL	AAV2	HLA-B07:02	HLA-B07:02	(6, 14, 15, 18, 19, 24)
IPQYGYLTL	AAV6, AAV9	HLA-B07:02	HLA-B07:02	(14, 19, 25)
LIDQYLYYL	AAV9	HLA-A02:01	HLA-A02:01, HLA-A02:02	(14, 17, 25)
QPAKKRLNF	AAV9	HLA-B07:02	HLA-B53	(14)
SQAVGRSSF	AAV6, AAV9	HLA-B15:01	HLA-B44	(14)
FPQSGVLIF	AAV2	HLA-B35:01	HLA-B51	(14)

Combined, we found that the immunopeptidomes of AAV capsids consist of peptides distributed along the entire protein, are arranged in single peptides as well as clusters and provide 59 new identifications that serve as potential epitopes.

### 3.3 More than 60% of the AAV HLA class I peptides are contained within clusters of HLA class II

**Figure 4:**
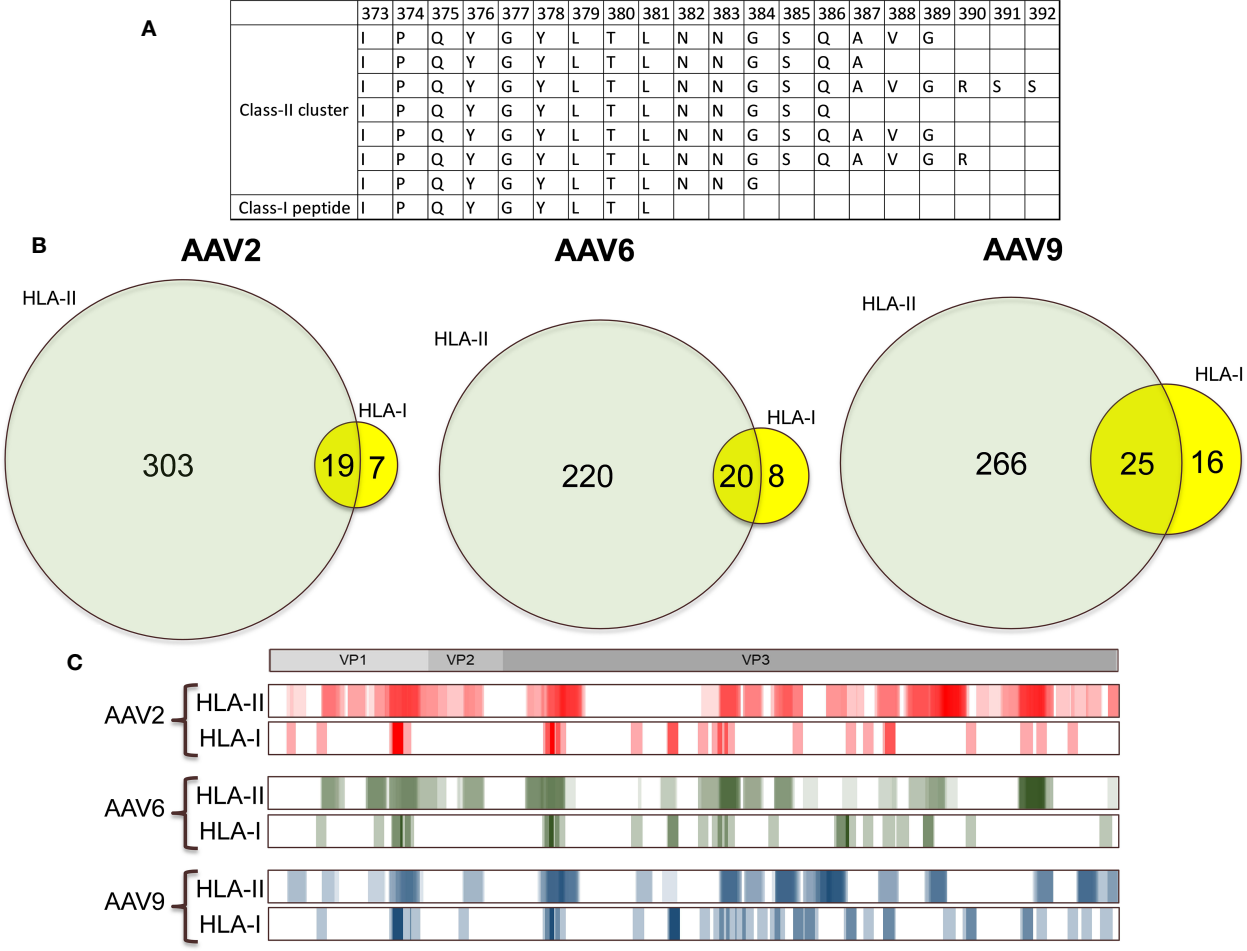
More than 60% of the AAV HLA class I peptides are contained within HLA class II clusters. **(A)** Representation of an HLA class I peptide contained within an HLA class II cluster. In this example we show the immunogenic epitope IPQYGYLTL fully contained within a cluster of HLA class II that have the similar sequences. **(B)** Venn diagrams showing the number of peptides that are unique and contained between HLA class II and HLA class I for each of the immunopeptidomes. **(C)** Heatmap comparison of the HLA class I and HLA class II immunopeptidomes. The heatmaps represent the sum of all the peptides that were displayed among the different cohorts in this study and in Brito-Sierra et al., 2022 (22). For ease of representation, the HLA class II heatmaps are shown in log2 scale.

Our recent publication reported the HLA class II immunopeptidomes of AAV2, AAV6, and AAV9 capsids, which showed significant sequence coverage of over 80% of the capsids (22). As similar peptide sequences can be presented by both HLA class I and class II molecules (26, 27), we aimed to compare the HLA class I and class II immunopeptidomes of the three AAV serotypes (**Figure 4A**). While the HLA class II immunopeptidome consisted of nested clusters of peptides of different length, the HLA class I peptides did not show much variation in peptide size. Thus, we searched for HLA class II clusters that contained the sequence of individual HLA class I peptides and identified 19, 20, and 25 HLA class I peptides that were contained within the HLA class II clusters for AAV2, AAV6, and AAV9, respectively (**Figure 4B**). These peptides were distributed along multiple regions of the capsid (**Figure 4C**). Overall, we observed that more than 60% of the AAV HLA class I peptides corresponded to sequences contained within the clusters identified in the HLA class II immunopeptidomes. These peptides hold significant importance as previous studies have suggested that peptides that are presented by both immune system molecules, result in the activation of polyfunctional cytokine responses in peripheral T cells (26).

### 3.4 Immunogenicity of the naturally displayed HLA class I peptides

We identified six naturally displayed peptides that are identical sequences to the AAV epitopes that had been described before (**Table 3**). Among these identical peptide sequences, we identified the highly validated AAV2 epitope VPQYGYLTL (6, 19) (**Table 3**, **Supplemental Table 2**) and its AAV6/AAV9 homolog IPQYGYLTL (6, 16, 19) (**Table 3**, **Supplemental Tables 3, 4**). We also identified the AAV9 LIDQYLYYL peptide, which has been validated with multimer/tetramer quantitative binding (17) (**Table 3**, **Supplemental Table 4**). Peptides VPQYGYLTL/IPQYGYLTL were associated with HLA-B07:01 and LIDQYLYYL was associated in HLA-A01:01 which are the same alleles that had been characterized in previous studies. In contrast, other peptides were associated with alleles different to the ones described before, suggesting that these immunodominant peptides are less restricted than initially described. For example, the peptide SQAVGRSSF was previously reported to be associated with the HLA-B44 allele. However, our analyses associated this peptide with the HLA-B15:01 allele. Notably, Hui et al., 2015 investigated the immunogenicity of the peptide SQAVGRSSF in a donor expressing both HLA-B15:01 and HLA-B44 alleles (14). Their bioinformatics analysis suggested that this peptide binds to the HLA-B44 allele. Nevertheless, as the donor also expressed the other allele, the possibility of the peptide also binding to HLA-B15:01 remains plausible, which aligns with our observations.

Despite having screened donors for the presumed correct allele, 12 peptides, which comprise half of all the epitopes reported in prior studies (**Supplemental Table 5**), were not detected. Among these, SADNNNSEY, a well-known AAV epitope with strong immunogenicity evidence (6, 14, 15, 19, 28), was absent in five donors carrying the HLA-A01:01 allele (donors B, D, H, J, and L) which is associated with this epitope. The absence of this epitope in our study despite ample sampling of appropriate donors suggests the involvement of a distinct processing and presentation mechanism. Mass spectrometry analysis of a synthesized peptide validated the identification of SADNNNSEY, indicating that the failure to detect this epitope in the MAPPs assay was not due to technical limitations, but rather a biological mechanism (**Supplemental Figure 2**). We also identified peptides that match sequences from published 9-mer epitopes, however the naturally processed peptides identified in our study were longer. In addition, these elongated sequences did not exhibit binding specificity to the same HLA allele as reported in previous studies (**Supplemental Table 6**). In summary, our results demonstrate that some of the naturally displayed HLA class I peptides of AAV serotypes match with previously identified epitopes, expand the range of HLA alleles that bind to AAV peptides, and introduce new epitopes with immunogenic potential.

### 3.5 Eleven HLA class I peptides are highly conserved among AAV serotypes

**Figure 5:**
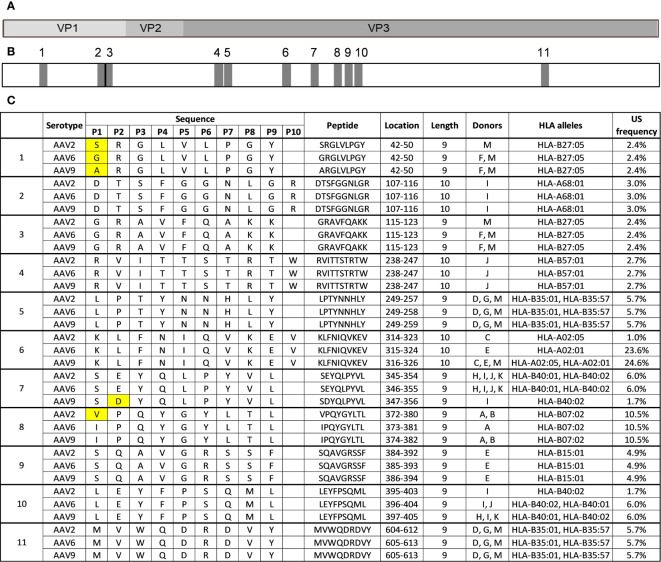
Eleven HLA class I peptides are highly conserved among AAV2, AAV6 and AAV9. **(A)** Diagram representation of the full-length sequence of VP1, highlighting the regions that correspond to VP1 unique, VP1/VP2 unique and VP3. **(B)** Diagram representing the position of each of the 11 conserved regions along the capsid protein as shown in A **(C)** Peptide information for the 11 peptides that are highly conserved among AAV serotypes. The first column labels the peptides by regions for each serotype and peptide. The peptide sequences are aligned with each other serotype. Yellow boxes represent amino acid residues that are different.

The high level of similarity between the protein sequences of AAV2, AAV6, and AAV9 capsids (>80% identity) means that highly conserved regions are likely present along the capsid of AAVs and may contribute to cross-reactivity between serotypes in the immunopeptidomes. In our study, we identified 11 highly conserved peptides along the capsid of AAVs that are present in the HLA class I immunopeptidomes of all three serotypes tested (**Figures 5A-C**). Eight of these peptides (2-6 and 9-11) had identical sequences among the three serotypes (**Figure 5C**), and peptides 5 and 11 were identified in the same set of multiple donors from all three serotypes, indicating a degree of promiscuity in these regions of interest.

Three peptides (1, 7, and 8) had variations in a single residue (**Figure 5C**). The conserved peptide 1 contained different amino acids (S in AAV2, G in AAV6, and A in AAV9) at the 4th residue but was identical for the rest of the sequence. All three variants are predicted to bind to the same HLA-B27:05 allele. The corresponding peptides from AAV6 and AAV9 were identified in the same subset of donors, while the AAV2 variant was only identified in a single donor, possibly due to minor affinity differences caused by the sequence difference. Conserved peptide 7 contained a change from E to D at the 2nd residue in the AAV9 variant, resulting in a reduced prevalence of display in the donor cohort. The AAV2 and AAV6 variants of conserved peptide 7, which have E at the 2nd residue, are predicted to be bound by both HLA-B40:01 and HLA-B40:02 alleles and were observed in the same 4 donors. The AAV9 variant, which has D at the 2nd residue, was only observed in one of those same four donors and only predicted to bind to HLA-B40:02 allele. This finding aligns with a prior investigation, which showed that the HLA-B40:02 allele has the capacity to present peptides containing either an E or D residue in the second position. In contrast, HLA-B40:01 predominantly permits peptides with an E residue at the second position, as depicted in **Supplemental Figure 3** (29).

Two of the highly conserved peptides, peptide 8 (VPQYGYLTL/IPQYGYLTL) and peptide 9 (SQAVGRSSF), correspond to previously described immunogenic epitopes. Additionally, two other conserved peptides correspond to clusters previously described in **Table 3** conserved peptide 2 (DTSFGGNLGR) is a member of cluster 1 and conserved peptide 4 (RVITTSTRTW) is a member of cluster 2. Taken together, these results highlight several HLA class I peptides of interest that could account for cross-reactivity across AAV serotypes and should be carefully examined.

## 4 Discussion

Our study aimed to describe the HLA class I immunopeptidomes of three AAVs (AAV2, AAV6, and AAV9), which are commonly used in clinical trials and approved therapies. To achieve this, we used a novel approach that involved identifying the HLA class I immunopeptidome of AAVs from intracellularly produced capsid in human cells. This method has several advantages over previous approaches, including: i) mRNA transfection, which leads to intracellular expression and peptide loading on HLA class I molecules that closely resembles HLA class I peptide loading via direct presentation, ii) using MDDCs as the expression host provided access to multiple alleles without the need for a different transfection method for each cell line, and iii) the MAPPs method allowed direct identification of peptides that are naturally processed and loaded on HLA class I molecules. Our study profiled 13 donors, covering more than 50% of the HLA class I diversity in the United States, accounting for 36 HLA class I alleles. The results of our study highlighted several key findings. First, we identified a total of 65 AAV peptides, six of which were identical to previously identified immunogenic epitopes using overlapping peptide pools. The remaining 59 peptides represent potential CD8 T cell epitopes that have not been previously reported. Second, we discovered 12 clusters that contained at least two peptides of different lengths. Third, more than 60% of the HLA class I peptides were contained within the HLA class II clusters. Finally, we found that 11 peptides were highly conserved among the three serotypes analyzed in this study.

Previous studies have identified CD8+ T cell epitopes of several AAV serotypes using one or more approaches, such as pools of overlapping peptides spanning the entire capsid sequence (14, 15, 30), bioinformatic algorithms predictions (31), MHC multimers and binding affinity assays (6, 17, 19). However, few of these studies have demonstrated that the peptides are naturally displayed by cells in HLA molecules. Our study expands on this work by describing the entire repertoire of naturally processed and displayed AAV-derived peptides from intracellular expression of AAV capsid. We identified 65 AAV capsid peptides, corresponding to 26 of AAV2, 28 of AAV6 and 41 of AAV9. Only 9% (6 peptides) correspond to epitopes that had been described before but three of these were predicted to bind a different allele than the ones described in previous studies. Differential allele binding prediction is probably the result of discrepancies in donor alleles and bioinformatic algorithms. For example, Hui et al., 2015 demonstrated that SQAVGRSSF is a binder of the HLA-B44 allele by testing it against six donors with said genotype, however the highest immunogenicity was observed in a subject with the HLA-B44 and HLA-B15:01 alleles (14). In our study, two donors (E and I) had the HLA-B44 allele, but we only identified SQAVGRSSF only in donor E, which also has the HLA-B15:01 allele. This would agree with the highest immunogenicity in the donor with the HLA-B15:01 allele described by Hui 2015 (14). Given that our analysis predicted that SQAVGRSSF binds HLA-B15:01, the binding repertoire for this epitope is now expanded to the two alleles. In addition, our results highlight that 91% of the immunopeptidomes (59 peptides) correspond to novel peptides not described before and should be evaluated in further immunogenicity assays. Early work has demonstrated that more than 80% of the HLA class I peptides of vaccinia virus identified by mass spectrometry are immunogenic (32), suggesting that many of the novel peptides that we identified in this study have the potential to elicit CD8+ T cell activation.

Most canonical peptide ligands for HLA class I molecules are 9–10 amino acids in length. However, non-canonical peptide ligands with more than 11 amino acids can also elicit dominant cytotoxic T lymphocyte responses, sometimes at the expense of overlapping shorter peptides (33). MAPPs analysis enabled the identification of these unusually long peptides that could be missed by epitope mapping studies that focus on 9-mer peptides. Among the 65 peptides that were identified in the current study, a subset of 15 peptides were observed to have 11-13 amino acids in length. Some of these peptides were distributed across the 12 clusters described in this study. Notably, some clusters exhibited peptides of varying lengths that were presented by the same donors and alleles. Such an observation is consistent with previous studies on influenza and may have significant implications for T cell responses (34). On the other hand, other clusters included peptides of varying lengths that were differentially presented by donors and alleles. These peptides may represent the activation of distinct T cell responses in various donors, given that HLA alleles have different preferences for peptide length and, hence, can influence T cell activation differently (35).

When evaluating pre-existing immunity, cross-reactivity among serotypes is another factor to consider. For instance, if AAV2 natural infections generated memory CD8+ T cells that recognize an identical epitope in AAV9, therapy using AAV9 could potentially activate and expand capsid-specific CD8+ T cells. Similar cellular responses due to identical epitopes from different sources have been described in various studies, not specific to AAVs (34, 36–41). Considering that at least 30% of the human population has been infected with one AAV serotype (42), and the capsid sequences of AAVs are well-preserved among serotypes, assessing cross-reactivity across serotypes is crucial. AAV2, AAV6, and AAV9, for example, share over 80% sequence identity. In our research, we discovered 11 HLA class I peptides that are conserved across the three AAV serotypes examined, with three of them corresponding to previously described immunogenic epitopes. Evaluating the immunogenic potential of the remaining conserved AAV peptides is essential to comprehend the degree of problematic cross-reactivity during clinical development using these vectors for gene delivery.

It is important to note that the approach used in this study to identify peptides presented on HLA class I molecules from intracellular protein expression may not fully reflect the HLA class I peptidome derived from AAV capsids during gene therapies, as AAVs are unable to express capsid protein intracellularly. The HLA class I peptidome from AAV capsids during gene therapies would be the result of cross-presentation and may differ from the peptides identified in this study. However, given that the MHC-multimer validated epitopes, VPQYGYLTL and IPQYGYLTL, have been tested on transduced cells with therapeutic AAV (6), one can argue that they correspond to peptides derived from cross-presentation. And given that we found these same peptides from intracellular protein expression, it seems reasonable to infer that at least the majority of the immunopeptidomes repertoire is similar with cross-presentation. Nevertheless, we did not observe 12 of the previous described epitopes (corresponding to about 50% of the published studies), including SADNNNSEY, which is a well characterized epitope and validated with multimer/tetramer qualitative binding assays (6, 14, 15, 19, 28). This is surprising, given that SADNNNSEY has been defined as an HLA-A01:01 binder, which is an allele present in 5 of the donors of our study (donors B, D, H, J and L). The lack of SADNNNSEY identification may potentially stem from technical limitations intrinsic to the MAPPs assay, such as the limited abundance of this peptide as well as ion interference. Further investigations utilizing targeted immunopeptidomics are necessary to ascertain the true absence of SADNNNSEY in transfected MDDCs. Nonetheless, it is also conceivable to explore the possibility of underlying biological factors. It is plausible to postulate that despite its immunogenic nature, the presentation of SADNNNSEY might not follow conventional direct presentation pathways but rather may occur through alternative mechanisms, such as cross-presentation. This assertation is supported by a study conducted by Wu et al. in 2019 (43), which investigated both direct and cross-presentation of influenza A HLA class I peptides in mouse cells and found varying levels of epitope abundance between the two modes of presentation (43). To date, cross-presentation has not been evaluated for any viral antigen in human cells, likely due to the technical challenges associated with these types of experiments, which require a large amount of concentrated viral particles. Future studies will need to address this matter by identifying clinically relevant cells and developing an optimal experimental platform to compare direct vs cross-presentation with mass spectrometry.

Early studies have shown that CD8+ T cell responses play a major role in anti-drug immunity of gene therapies (6, 7, 44). Therefore, regulatory agencies are encouraging the monitoring of CD8+ T cell responses as part of the immunogenicity risk assessment of AAVs. Currently, the CD8+ T cell assays are performed using overlapping peptides spanning the entire capsid of AAVs. This method carries the risk of activating cells with peptides that would not be naturally presented, leading to the potential identification of false positive. Therefore, our study sets the stage for alternative pools with naturally displayed peptides. Future studies will determine the magnitude of the CD8+ T cell activation with the naturally displayed HLA class I peptides and establish the risk of cross-reactivity among serotypes. These peptides will provide the basis for assessment of cellular responses against gene therapies in clinical trials.

## Data availability statement

The datasets presented in this study can be found in online repositories. The names of the repository/repositories and accession number(s) can be found below: https://massive.ucsd.edu/ProteoSAFe/dataset.jsp?task=fd8c46702b004d52897ac75345a3b599.

## Author contributions

Experimental design, method development, investigation, analysis, data curation, visualization, editing, writing – original draft: CB-S. Experimental design, method development, writing – review & editing: ML. Supervision, analysis, writing– review & editing: RS. Supervision, analysis, writing – review & editing: LM. All authors contributed to the article and approved the submitted version.

## References

[1] Wang D Tai PWL Gao G. Adeno-associated virus vector as a platform for gene therapy delivery. Nat Rev Drug Discovery (2019) 18(5):358–78. doi: 10.1038/s41573-019-0012-9 PMC6927556 30710128

[2] Li C Samulski RJ. Engineering adeno-associated virus vectors for gene therapy. Nat Rev Genet (2020) 21(4):255–72. doi: 10.1038/s41576-019-0205-4 32042148

[3] Dalwadi DA Calabria A Tiyaboonchai A Posey J Naugler WE Montini E. AAV integration in human hepatocytes. Mol Ther (2021) 29(10):2898–909. doi: 10.1016/j.ymthe.2021.08.031 PMC8531150 34461297

[4] Cring MR Sheffield VC. Gene therapy and gene correction: targets, progress, and challenges for treating human diseases. Gene Ther (2022) 29(1-2):3–12. doi: 10.1038/s41434-020-00197-8 33037407

[5] Mullard A. FDA approves first haemophilia B gene therapy. Nat Rev Drug Discovery (2023) 22(1):7. doi: 10.1038/d41573-022-00199-8 36460866

[6] Mingozzi F Maus MV Hui DJ Sabatino DE Murphy SL Rasko JE. CD8(+) T-cell responses to adeno-associated virus capsid in humans. Nat Med (2007) 13(4):419–22. doi: 10.1038/nm1549 17369837

[7] Manno CS Pierce GF Arruda VR Glader B Ragni M Rasko JJ. Successful transduction of liver in hemophilia by AAV-Factor IX and limitations imposed by the host immune response. Nat Med (2006) 12(3):342–7. doi: 10.1038/nm1358 16474400

[8] Basner-Tschakarjan E Mingozzi F. Cell-mediated immunity to AAV vectors, evolving concepts and potential solutions. Front Immunol (2014) 5:350. doi: 10.3389/fimmu.2014.00350 25101090 PMC4107954

[9] Hamilton BA Wright JF. Challenges posed by immune responses to AAV vectors: Addressing root causes. Front Immunol (2021) 12:675897. doi: 10.3389/fimmu.2021.675897 34084173 PMC8168460

[10] Finn JD Nichols TC Svoronos N Merricks EP Bellenger DA Zhou S. The efficacy and the risk of immunogenicity of FIX Padua (R338L) in hemophilia B dogs treated by AAV muscle gene therapy. Blood. (2012) 120(23):4521–3. doi: 10.1182/blood-2012-06-440123 PMC3512231 22919027

[11] Ronzitti G Gross DA Mingozzi F. Human immune responses to adeno-associated virus (AAV) vectors. Front Immunol (2020) 11:670. doi: 10.3389/fimmu.2020.00670 32362898 PMC7181373

[12] Ertl HCJ. Preclinical models to assess the immunogenicity of AAV vectors. Cell Immunol (2019) 342:103722. doi: 10.1016/j.cellimm.2017.11.006 29195742

[13] Madsen D Cantwell ER O'Brien T Johnson PA Mahon BP. Adeno-associated virus serotype 2 induces cell-mediated immune responses directed against multiple epitopes of the capsid protein VP1. J Gen virology (2009) 90(Pt 11):2622–33. doi: 10.1099/vir.0.014175-0 PMC2885037 19641045

[14] Hui DJ Edmonson SC Podsakoff GM Pien GC Ivanciu L Camire RM. AAV capsid CD8+ T-cell epitopes are highly conserved across AAV serotypes. Mol Ther Methods Clin Dev (2015) 2:15029. doi: 10.1038/mtm.2015.29 26445723 PMC4588448

[15] Hui DJ Basner-Tschakarjan E Chen Y Davidson RJ Buchlis G Yazicioglu M. Modulation of CD8+ T cell responses to AAV vectors with IgG-derived MHC class II epitopes. Mol Ther (2013) 21(9):1727–37. doi: 10.1038/mt.2013.166 PMC3776637 23857231

[16] Mingozzi F Meulenberg JJ Hui DJ Basner-Tschakarjan E Hasbrouck NC Edmonson SA. AAV-1-mediated gene transfer to skeletal muscle in humans results in dose-dependent activation of capsid-specific T cells. Blood. (2009) 114(10):2077–86. doi: 10.1182/blood-2008-07-167510 PMC2744569 19506302

[17] Vandamme C Xicluna R Hesnard L Devaux M Jaulin N Guilbaud M. Tetramer-based enrichment of preexisting anti-AAV8 CD8(+) T cells in human donors allows the detection of a T(EMRA) subpopulation. Front Immunol (2019) 10:3110. doi: 10.3389/fimmu.2019.03110 32038634 PMC6990124

[18] Finn JD Hui D Downey HD Dunn D Pien GC Mingozzi F. Proteasome inhibitors decrease AAV2 capsid derived peptide epitope presentation on MHC class I following transduction. Mol Ther (2010) 18(1):135–42. doi: 10.1038/mt.2009.257 PMC2839204 19904235

[19] Pien GC Basner-Tschakarjan E Hui DJ Mentlik AN Finn JD Hasbrouck NC. Capsid antigen presentation flags human hepatocytes for destruction after transduction by adeno-associated viral vectors. J Clin Invest (2009) 119(6):1688–95. doi: 10.1172/JCI36891 PMC2689109 19436115

[20] Karle AC. Applying MAPPs assays to assess drug immunogenicity. Front Immunol (2020) 11:698. doi: 10.3389/fimmu.2020.00698 32373128 PMC7186346

[21] Knierman MD Lannan MB Spindler LJ McMillian CL Konrad RJ Siegel RW. The human leukocyte antigen class II immunopeptidome of the SARS-CoV-2 spike glycoprotein. Cell Rep (2020) 33(9):108454. doi: 10.1016/j.celrep.2020.108454 33220791 PMC7664343

[22] Brito-Sierra CA Lannan MB Siegel RW Malherbe LP. The HLA class-II immunopeptidomes of AAV capsids proteins. Front Immunol (2022) 13:1067399. doi: 10.3389/fimmu.2022.1067399 36605211 PMC9807805

[23] Kaabinejadian S Barra C Alvarez B Yari H Hildebrand WH Nielsen M. Accurate MHC motif deconvolution of immunopeptidomics data reveals a significant contribution of DRB3, 4 and 5 to the total DR immunopeptidome. Front Immunol (2022) 13:835454. doi: 10.3389/fimmu.2022.835454 35154160 PMC8826445

[24] Martino AT Basner-Tschakarjan E Markusic DM Finn JD Hinderer C Zhou S. Engineered AAV vector minimizes in *vivo* targeting of transduced hepatocytes by capsid-specific CD8+ T cells. Blood. (2013) 121(12):2224–33. doi: 10.1182/blood-2012-10-460733 PMC3606062 23325831

[25] Gernoux G Gruntman AM Blackwood M Zieger M Flotte TR Mueller C. Muscle-directed delivery of an AAV1 vector leads to capsid-specific T cell exhaustion in nonhuman primates and humans. Mol Ther (2020) 28(3):747–57. doi: 10.1016/j.ymthe.2020.01.004 PMC7054721 31982038

[26] Fujiwara K Shao Y Niu N Zhang T Herbst B Henderson M. Direct identification of HLA class I and class II-restricted T cell epitopes in pancreatic cancer tissues by mass spectrometry. J Hematol Oncol (2022) 15(1):154. doi: 10.1186/s13045-022-01373-6 36284347 PMC9597957

[27] Nagler A Kalaora S Barbolin C Gangaev A Ketelaars SLC Alon M. Identification of presented SARS-CoV-2 HLA class I and HLA class II peptides using HLA peptidomics. Cell Rep (2021) 35(13):109305. doi: 10.1016/j.celrep.2021.109305 34166618 PMC8185308

[28] Kared H Redd AD Bloch EM Bonny TS Sumatoh H Kairi F. SARS-CoV-2-specific CD8+ T cell responses in convalescent COVID-19 individuals. J Clin Invest (2021) 131(5):e145476. doi: 10.1172/JCI145476 33427749 PMC7919723

[29] Sarkizova S Klaeger S Le PM Li LW Oliveira G Keshishian H. A large peptidome dataset improves HLA class I epitope prediction across most of the human population. Nat Biotechnol (2020) 38(2):199–209. doi: 10.1038/s41587-019-0322-9 31844290 PMC7008090

[30] Hakkim A Fuchs TA Martinez NE Hess S Prinz H Zychlinsky A. Activation of the Raf-MEK-ERK pathway is required for neutrophil extracellular trap formation. Nat Chem Biol (2011) 7(2):75–7. doi: 10.1038/nchembio.496 21170021

[31] Bing SJ Justesen S Wu WW Sajib AM Warrington S Baer A. Differential T cell immune responses to deamidated adeno-associated virus vector. Mol Ther - Methods Clin Dev (2022) 24:255–67. doi: 10.1016/j.omtm.2022.01.005 PMC8829777 35211638

[32] Croft NP Smith SA Pickering J Sidney J Peters B Faridi P. Most viral peptides displayed by class I MHC on infected cells are immunogenic. Proc Natl Acad Sci U S A. (2019) 116(8):3112–7. doi: 10.1073/pnas.1815239116 PMC6386720 30718433

[33] Burrows JM Bell MJ Brennan R Miles JJ Khanna R Burrows SR. Preferential binding of unusually long peptides to MHC class I and its influence on the selection of target peptides for T cell recognition. Mol Immunol (2008) 45(6):1818–24. doi: 10.1016/j.molimm.2007.09.026 17981331

[34] Assmus LM Guan J Wu T Farenc C Sng XYX Zareie P. Overlapping peptides elicit distinct CD8(+) T cell responses following influenza A virus infection. J Immunol (2020) 205(7):1731–42. doi: 10.4049/jimmunol.2000689 PMC7511415 32868409

[35] Trolle T McMurtrey CP Sidney J Bardet W Osborn SC Kaever T. The length distribution of class I-restricted T cell epitopes is determined by both peptide supply and MHC allele-specific binding preference. J Immunol (2016) 196(4):1480–7. doi: 10.4049/jimmunol.1501721 PMC4744552 26783342

[36] Mishto M Mansurkhodzhaev A Rodriguez-Calvo T Liepe J. Potential Mimicry of Viral and Pancreatic beta Cell Antigens Through Non-Spliced and cis-Spliced Zwitter Epitope Candidates in Type 1 Diabetes. Front Immunol (2021) 12:656451. doi: 10.3389/fimmu.2021.656451 33936085 PMC8082463

[37] Mansurkhodzhaev A Barbosa CRR Mishto M Liepe J. Proteasome-generated cis-spliced peptides and their potential role in CD8(+) T cell tolerance. Front Immunol (2021) 12:614276. doi: 10.3389/fimmu.2021.614276 33717099 PMC7943738

[38] Sangewar N Waghela SD Yao J Sang H Bray J Mwangi W. Novel potent IFN-gamma-inducing CD8(+) T cell epitopes conserved among diverse bovine viral diarrhea virus strains. J Immunol (2021) 206(8):1709–18. doi: 10.4049/jimmunol.2001424 33762324

[39] Zhu H Liu X Wu Y He Y Zheng H Liu H. Identification of a neutralizing linear epitope within the VP1 protein of coxsackievirus A10. Virol J (2022) 19(1):203. doi: 10.1186/s12985-022-01939-3 36457099 PMC9714398

[40] Luo WR Wu XM Wang W Yu JL Chen QQ Zhou X. Novel coronavirus mutations: Vaccine development and challenges. Microb Pathog (2022) 173(Pt A):105828. doi: 10.1016/j.micpath.2022.105828 36243381 PMC9561474

[41] Gordin M Philip H Zilberberg A Gidoni M Margalit R Clouser C. Breast cancer is marked by specific, Public T-cell receptor CDR3 regions shared by mice and humans. PloS Comput Biol (2021) 17(1):e1008486. doi: 10.1371/journal.pcbi.1008486 33465095 PMC7846026

[42] Aronson SJ Veron P Collaud F Hubert A Delahais V Honnet G. Prevalence and relevance of pre-existing anti-adeno-associated virus immunity in the context of gene therapy for crigler-najjar syndrome. Hum Gene Ther (2019) 30(10):1297–305. doi: 10.1089/hum.2019.143 PMC6763963 31502485

[43] Wu T Guan J Handel A Tscharke DC Sidney J Sette A. Quantification of epitope abundance reveals the effect of direct and cross-presentation on influenza CTL responses. Nat Commun (2019) 10(1):2846. doi: 10.1038/s41467-019-10661-8 31253788 PMC6599079

[44] Chen J Wu Q Yang P Hsu HC Mountz JD. Determination of specific CD4 and CD8 T cell epitopes after AAV2- and AAV8-hF. IX Gene Ther Mol Ther (2006) 13(2):260–9. doi: 10.1016/j.ymthe.2005.10.006 16324888

