# Supplementary material for: The HLA class I immunopeptidomes of AAV capsid proteins
Source: Front Immunol. 2023 Aug 16;14:1212136. doi: 10.3389/fimmu.2023.1212136 (PMC10469481; doi:10.3389/fimmu.2023.1212136)
Supplement: Supplementary file 1 [file DataSheet_1.pdf]

Document S1. Figures S1-S3, Tables S1-S6

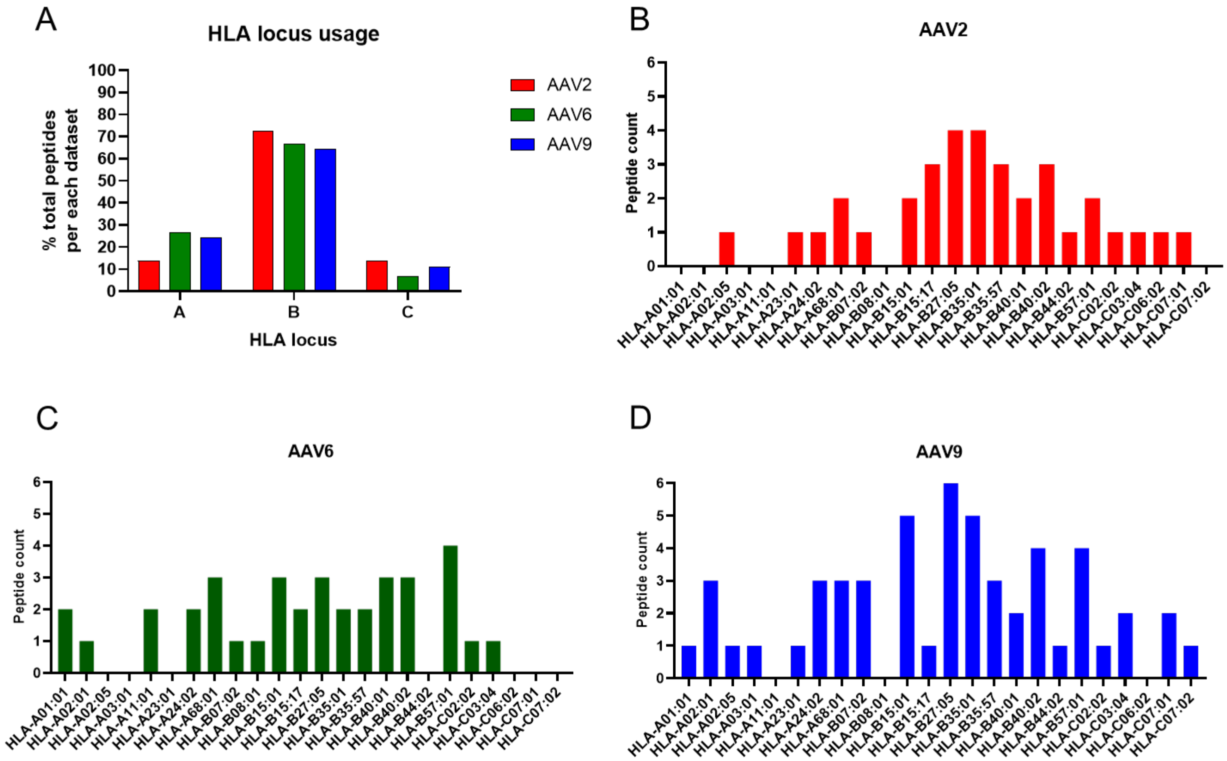

Figure S1. HLA allele frequency and predicted binding. A. HLA locus usage represented as the percentage of total peptide numbers of each AAV dataset. (B-D). Number of peptides predicted to bind to each allele (x-axis) for AAV2 (B), AAV6 (C) or AAV9 (D)

A

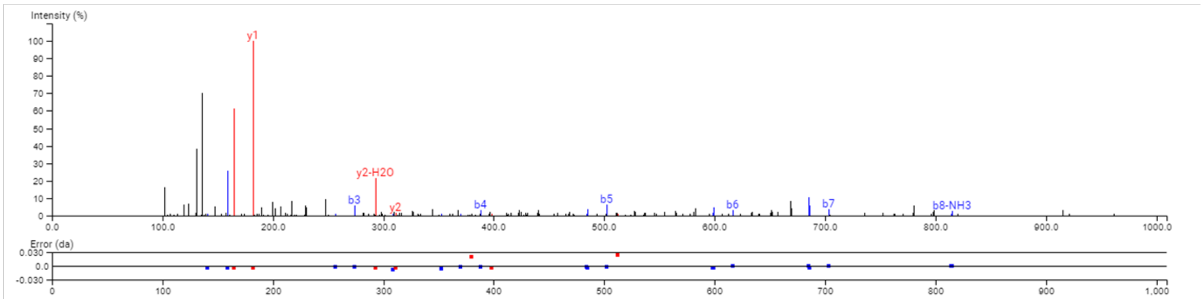

B

C

#	b	b-H2O	b-NH3	b(2+)	Seq	y	y-H2O	y-NH3	y(2+)	#
1	88.04	70.03	71.01	44.52	S					9
2	159.08	141.07	142.05	80.04	A	926.35	908.34	909.32	463.67	8
3	274.10	256.09	257.08	137.55	D	855.31	837.30	838.28	428.16	7
4	388.14	370.13	371.12	194.57	N	740.28	722.27	723.26	370.64	6
5	502.19	484.18	485.16	251.59	N	626.24	608.23	609.21	313.62	5
6	616.23	598.22	599.21	308.62	N	512.17	494.19	495.17	256.60	4
7	703.26	685.25	686.24	352.14	S	398.16	380.12	381.13	199.58	3
8	832.31	814.29	815.28	416.65	E	311.12	293.11	294.10	156.06	2
9					Y	182.08	164.07	165.05	91.54	1

Figure S2. Graphical representation of the spectra of SADNNNSEY. 5 µL of synthesized peptide were injected in the QE-HFX mass spectrometer at a concentration of 400 nM. The instrument settings and the analysis on PEAKS was performed as described in the methods section. (A) Spectra representation where the y-axis is the intensity shown in percentage and the x-axis is the m/z. (B) Graphical representation showing the difference between the measured mass of an ion and its expected or theoretical mass. The m/z ratio is displayed on the x-axis and the error is shown on the y-axis in Daltons. Each matched fragment ion is represented by a dot. (C) Ion table showing the calculated mass of possible fragment ions based on the ion types. The mass value of a detected fragment ion in the spectrum is depicted in color. The N-terminal ions are designated by blue, and the C-terminal ions are designated by red. A fragment ion is considered found if a matching peak is detected within the mass error tolerance range.

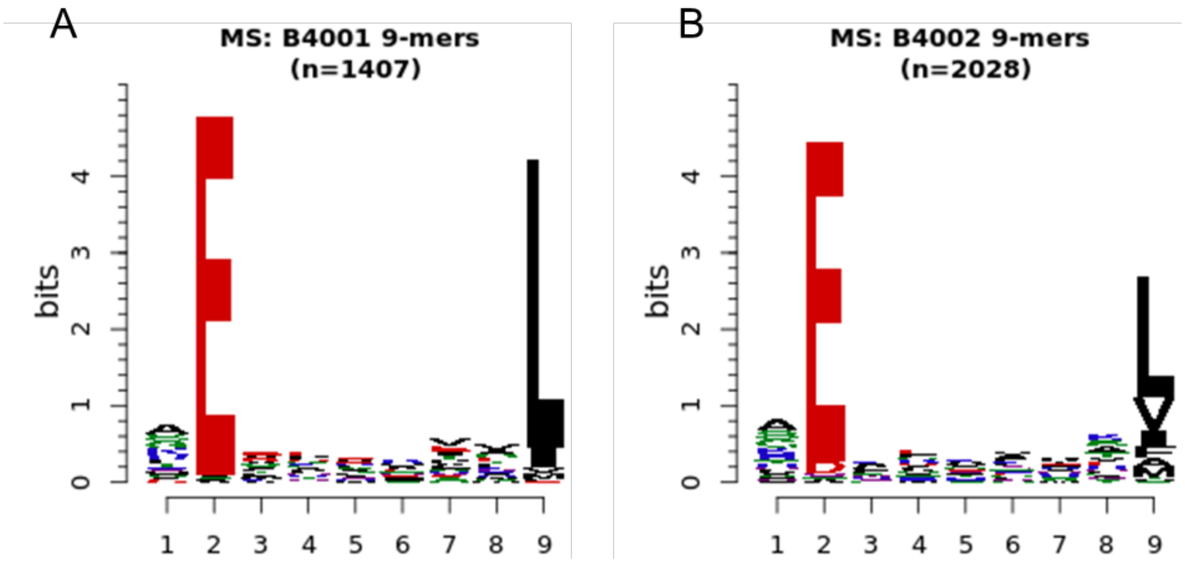

Figure S3. Sequence logos of HLA-B40:01 and HLA-B:40:02. The sequence logos were obtained from http://hlathena.tools/. The sequence logos visually represent the amino acid preferences at specific positions for HLA-B40:01 (A) and HLA-B40:02 (B). In the case of HLA-B40:01, peptides with an E at position 2 are predominantly presented, while peptides with a D at that position are rare, constituting only 0.26% of the peptides. On the other hand, HLA-B40:02 displays a higher frequency of presenting peptides with a D at position 2, which occurs approximately 1.87% of the time (Sarkizova *et al* 2019).

Supplemental table 1. Peptide numbers across donors and serotypes

Donor	Total unique peptide count	AAV2	AAV6	AAV9
A	2826	2	1	4
B	2211	1	2	2
C	3188	2	2	4
D	2358	3	5	3
E	3237	2	4	6
F	1713	2	4	7
G	4408	3	4	4
H	3163	2	4	5
I	3869	6	6	8
J	3522	5	9	9
K	2410	1	4	2
L	2964	3	3	2
M	3208	9	6	13

Supplemental table 2. AAV2 capsid-derived HLA class I peptides

Peptide	Length	Start	End	HLA allele	Donor	Number of positive donors per MHC allele	Immunogenicity evidence	Reference	Previous reported MHC allele
SEGIRQWW	8	16	23	HLA-B44:02	I	1/2	-	-	-
SRGLVLPGY	9	42	50	HLA-B27:05	M	1/2	-	-	-
KEDTSFGGNL	10	105	114	HLA-B40:01	J	1/3	-	-	-
DTSFGGNLGR	10	107	116	HLA-A68:01	I	1/1	-	-	-
TSFGGNLGR	9	108	116	HLA-A68:01	I	1/1	-	-	-
GRAVFQAKK	9	115	123	HLA-B27:05	M	1/2	-	-	-
RVITTSTRTW	10	238	247	HLA-B57:01	J	1/1	-	-	-
ITTSTRTW	8	240	247	HLA-B57:01	J	1/1	-	-	-
TRTWALPTY	9	244	252	HLA-B27:05, HLA-C07:01	F, M	1/2, 1/5	-	-	-
LPTYNNHLY	9	249	257	HLA-B35:01, HLA-B35:57	D, M, G	2/2, 1/1	-	-	-
KLFNIQVKEV	10	314	323	HLA-A02:05	C	1/1	-	-	-
DSEYQLPYVL	10	345	354	HLA-B40:02	I	1/1	-	-	-
SEYQLPYVL	9	346	354	HLA-B40:01, HLA-B40:02	H, I, J, K	3/3, 1/1	-	-	-
VPQYGYLTL	9	372	380	HLA-B07:02	A, B	2/3	Yes	(6, 14, 19)	HLA-B07:02
SQAVGRSSF	9	384	392	HLA-B15:01	E	1/2	Yes	(14)	HLA-B44
RSSFYCLEY	9	389	397	HLA-B15:17	L	1/1	-	-	-
LEYFPSQML	9	395	403	HLA-B40:02	I	1/1	-	-	-
TTTQSRLQF	9	454	462	HLA-B15:17	L	1/1	-	-	-
YSWTGATKY	9	500	508	HLA-B15:17, HLA-C02:02	L, M	1/1, 1/3	-	-	-
SLVNPGPAM	9	515	523	HLA-C03:04	H, J	2/5	-	-	-
KFFPQSGVLIF	11	532	542	HLA-A23:01, HLA-A24:02	A, F	1/1, 1/1	-	-	-
FPQSGVLIF	9	534	542	HLA-B35:01	M	1/2	Yes	(14)	HLA-B51
MVWQDRDVY	9	604	612	HLA-B35:01, HLA-B35:57	D, M, G	2/2, 1/1	-	-	-
TPVPANPSTTF	11	651	661	HLA-B35:01, HLA-B35:57	D, M, G	2/2, 1/1	-	-	-
KFASFITQY	9	665	673	HLA-B15:01, HLA-C06:02	C, E	1/2, 1/3	-	-	-
KRWNPEIQY	9	692	700	HLA-B27:05	M	1/2	-	-	-

Peptides predicted to bind different HLA locus were separated to properly calculate the US frequency

Supplemental table 3. AAV6 capsid-derived HLA class I peptides

Peptide	Length	Start	End	MHC allele	Donor	Number of positive donors per MHC allele	Immunogenicity evidence	Reference	Previous reported MHC allele
GRGLVLPGY	9	42	50	HLA-B27:05	F, M	2/2	-	-	-
RYNHADAEF	9	92	100	HLA-A24:02	F	1/1	-	-	-
DTSFGGNLGR	10	107	116	HLA-A68:01	I	1/1	-	-	-
TSFGGNLGR	9	108	116	HLA-A68:01	I	1/1	-	-	-
GRAVFQAKK	9	115	123	HLA-B27:05	F, M	2/2	-	-	-
FQAKKRVL	8	119	126	HLA-B08:01	H	1/3	-	-	-
RVITTSTRTW	10	238	247	HLA-B57:01	J	1/1	-	-	-
ITTSTRTW	8	240	247	HLA-B57:01	J	1/1	-	-	-
TRTWALPTY	9	244	252	HLA-B27:05	M	1/2	-	-	-
LPTYNNHLY	9	249	257	HLA-B35:01, HLA-B35:57	D,M, G	2/3, 1/1	-	-	-
KLFNIQVKEV	10	315	324	HLA-A02:01	E	1/7	-	-	-
DSEYQLPYVL	10	346	355	HLA-B40:02	I	1/1	-	-	-
SEYQLPYVL	9	347	355	HLA-B40:01, HLA-B40:02	J, K, H, I	3/3, 1/1	-	-	-
IPQYGYLTL	9	373	381	HLA-B07:02	A	1/3	Yes	(14, 19)	HLA-B07:02
SQAVGRSSF	9	385	393	HLA-B15:01	E	1/2	Yes	(14)	HLA-B44
RSSFYCLEY	9	390	398	HLA-B15:17	L	1/1	-	-	-
LEYFPSQML	9	396	404	HLA-B40:02, HLA-B40:01	I, J	1/1, 1/3	-	-	-
LMNPLIDQY	9	434	442	HLA-B15:01	E	1/2	-	-	-
KTKTDNNNSNFTW	13	491	503	HLA-B57:01	J	1/1	-	-	-
KTDNNNSNFTW	11	493	503	HLA-B57:01	J	1/1	-	-	-
FTWTGASKY	9	501	509	HLA-B15:17	L	1/1	-	-	-
SIINPGTAM	9	516	524	HLA-C03:04	C, E, H, J, K	5/5	-	-	-
KFFPMSGVMIF	11	533	543	HLA-A24:02	F	1/1	-	-	-
KESAGASNTAL	11	545	555	HLA-B40:01	J	1/3	-	-	-
ATNPVATER	9	568	576	HLA-A11:01, HLA-A68:01	D, G, I, K	3/4, 1/1	-	-	-
ATNPVATERF	10	568	577	HLA-A01:01, HLA-A11:01, HLA-B15:01, HLA-C02:02	B,C, D, G, K, M	2/5, 2/4, 1/2, 1/3	-	-	-
MVWQDRDVY	9	605	613	HLA-B35:01, HLA-B35:57	D,M, G	2/3, 1/1	-	-	-
YTEPRPIGTRY	11	721	731	HLA-A01:01	B, D, H, J, L	5/5	-	-	-

Supplemental table 4. AAV9 capsid-derived HLA class I peptides

Peptide	Length	Start	End	HLA allele	Donor	Number of positive donors per MHC allele	Immunogenicity evidence	Reference	Previous reported MHC allele
ARGLVLPGY	9	42	50	HLA-B27:05	F, M	2/2	-	-	-
KEDTSFGGNL	10	105	114	HLA-B40:01	H, J, K	3/3	-	-	-
DTSFGGNLGR	10	107	116	HLA-A68:01	I	1/1	-	-	-
TSFGGNLGR	9	108	116	HLA-A68:01	I	1/1	-	-	-
GRAVFQAKK	9	115	123	HLA-B27:05	F, M	2/2	-	-	-
KRLLEPLGL	9	123	131	HLA-B27:05	F	1/2	-	-	-
QPAKKRLNF	9	165	173	HLA-B07:02	B, G	2/3	Yes	(14)	HLA-B53
RVITTSTRTW	10	238	247	HLA-B57:01	J	1/1	-	-	-
ITTSTRTW	8	240	247	HLA-B57:01	J	1/1	-	-	-
TRTWALPTY	9	244	252	HLA-B27:05, HLA-C07:01	F, M	1/2, 1/5	-	-	-
LPTYNNHLY	9	249	257	HLA-B35:01, HLA-B35:57	D, M, G	2/2, 1/1	-	-	-
KLFNIQVKEV	10	316	325	HLA-A02:05, HLA-A02:01	C, E, M	1/1, 1/7	-	-	-
TDSDYQLPYVL	11	346	356	Undefined	I	-	-	-	-
DSDYQLPYVL	10	347	356	Undefined	I	-	-	-	-
SDYQLPYVL	9	348	356	HLA-B40:02	I	1/1	-	-	-
IPQYGYLTL	9	374	382	HLA-B07:02	A, B	2/3	Yes	(14, 19)	HLA-B07:02
SQAVGRSSF	9	386	394	HLA-B15:01	E	1/2	Yes	(14)	HLA-B44
RSSFYCLEY	9	391	399	HLA-B15:17	L	1/1	-	-	-
LEYFPSQML	9	397	405	HLA-B40:01, HLA-B40:02	H, I, K	2/3, 1/1	-	-	-
LRTGNNFQF	9	405	413	HLA-B27:05	M	1/2	-	-	-
FQFSYEFENV	10	411	420	HLA-A02:01	H	1/7	-	-	-
SSYAHSQSL	9	424	432	HLA-C03:04	H, J	2/5	-	-	-
LMNPLIDQY	9	435	443	HLA-B15:01	E	1/2	-	-	-
LIDQYLYYL	9	439	447	HLA-A02:01	H, J	2/7	Yes	(14, 17)	HLA-A02:01
GQNQQTLKF	9	455	463	HLA-B15:01	C, E	2/2	-	-	-
GQNQQTLKF	9	455	463	HLA-C07:01, HLA-C02:02	L, M	1/5, 1/3	-	-	-
SVAGPSNMAVQGR	13	464	476	HLA-A68:01	I	1/1	-	-	-
VAGPSNMAV	9	465	473	HLA-C03:04	J	1/5	-	-	-
SEFAWPGASSW	11	499	509	HLA-B44:02	E, I	2/2	-	-	-
FAWPGASSW	9	501	509	HLA-B57:01, HLA-B35:01	J, M	1/1, 1/2	-	-	-
SLMNPGPAM	9	516	524	HLA-B15:01	C	1/2	-	-	-
RFFPLSGSLI	10	533	542	HLA-A24:02	F	1/1	-	-	-
RFFPLSGSLIF	11	533	543	HLA-A23:01, HLA-A24:02	A, F	1/1, 1/1	-	-	-
QSAQAQAQTGW	11	585	595	HLA-B57:01	J	1/1	-	-	-
LPGMVWQDRDVY	12	602	613	HLA-B35:01	M	1/2	-	-	-
MVWQDRDVY	9	605	613	HLA-B35:01, HLA-B35:57	D, M, G	2/2, 1/1	-	-	-
TPVPADPPTAF	11	652	662	HLA-B35:01, HLA-B35:57	D, M, G	2/2, 1/1	-	-	-
VPADPPTAF	9	654	662	HLA-B07:02	A	1/3	-	-	-
KLNSFITQY	9	666	674	HLA-A03:01, HLA-B15:01	C, E, M	1/2, 2/2	-	-	-
KRWNPEIQY	9	693	701	HLA-B27:05	M	1/2	-	-	-
YYKSNNVEF	9	705	713	HLA-A24:02, HLA-C07:02	A, F	1/1, 1/3	-	-	-
YS**EPRPIGTRY**	11	721	731	HLA-A01:01	J	1/5	-	-	-

Peptides TDSDYQLPYVL and DSDYQLPYVL were predicted to bind HLA-B40:02. However, the Rank score was >2, which is the threshold for accurate prediction of HLA class I alleles in the MHCMotifDecon software. Therefore, their HLA allele for these two peptides remains undefined.

Supplemental table 5. Epitopes described before but not identified in our study.

Epitope	Serotype	HLA allele(s)	Number of donors in our study with the HLA allele	References
SADNNNSEY	AAV2	HLA-A01:01	5	(6, 14, 15, 19, 26)
KYLGPGNGL	AAV9	HLA-A02:01	7	(45)
		HLA-A02:03	0	
		HLA-A23:01	1	
		HLA-B15:01	2	
TLNNGSQA	AAV2<br>AAV6	HLA-A02:01	7	(45)
		HLA-A02:03	0	
		HLA-B15:01	2	
		HLA-B44:02	2	
		HLA-A02:02	0	
YLSKTINGS	AAV9	HLA-A02:01	7	(45)
		HLA-A02:03	0	
		HLA-A11:01	4	
		HLA-B44:02	2	
YHLNGRDSL	AAV2	HLA-B15:01	2	(14)
YNLNGRESI	AAV6	HLA-B15:10	2	(14)
ALNGRNSLM	AAV9	HLA-A02:01	7	(45)
		HLA-A02:03	0	
		HLA-A03:01	2	
		HLA-A11:01	4	
		HLA-B15:01	2	
		HLA-B44:02	2	
FPMSGVMIF	AAV6	HLA-B51	1	(14, 44)
VPANPPAEF	AAV6	HLA-B51	1	(14, 44)
FTVDNNGLY	AAV6	HLA-A01:01	5	(45)
		HLA-B15:01	2	
TTSTRTWAL	AAV2<br>AAV6<br>AAV9	HLA-B08:01	3	(14, 44)
LDRLMNPLI	AAV2<br>AAV6<br>AAV9	HLA-A02:01	7	(14)

Supplemental table 6. Naturally processed HLA class I peptides with longer sequence than the epitopes described in previous studies

MAPPs peptide identified in our study	Serotype where MAPPs peptide was found	HLA alleles identified in our study	Epitope described in previous studies	HLA alleles described in previous studies	References
KFFPQSGVLIF	AAV2	HLA-A23:01, HLA-A24:02	FPQSGVLIF	HLA-B51	(14)
KTKTDNNNSNFTW	AAV6	HLA-B57:01	KTDNNNSNF	HLA-A01:01	(14, 44)
KTDNNNSNFTW	AAV6	HLA-B57:01			
TPVPANPSTTF	AAV2	HLA-B35:01, HLA-B35:57	VPANPSTTF	HLA-B51	(14)
YTEPRPIGTRY	AAV6	HLA-A01:01	EPRPIGTRY	HLA-B15:10	(14, 16)
YSEPRPIGTRY	AAV9	HLA-A01:01			
